# *Rickettsia helvetica* in *Dermacentor reticulatus* Ticks

**DOI:** 10.3201/eid1501.080815

**Published:** 2009-01

**Authors:** Marinko Dobec, Dragutin Golubic, Volga Punda-Polic, Franz Kaeppeli, Martin Sievers

**Affiliations:** Medizinische Laboratorien Dr F. Kaeppeli, Zurich, Switzerland (M. Dobec, F. Kaeppeli); University of Split School of Medicine, Split, Croatia (M. Dobec, V. Punda-Polic); County Hospital, Cakovec, Croatia (D. Golubic); Split University Hospital, Split (V. Punda-Polic); Zurich University of Applied Sciences, Waedenswil, Switzerland (M. Sievers)

**Keywords:** Dermacentor reticulatus, Rickettsia helvetica, spotted fever group rickettsiae, ticks, molecular analysis, dispatch

## Abstract

We report on the molecular evidence that *Dermacentor reticulatus* ticks in Croatia are infected with *Rickettsia helvetica* (10%) or *Rickettsia slovaca* (2%) or co-infected with both species (1%). These findings expand the knowledge of the geographic distribution of *R. helvetica* and *D. reticulatus* ticks.

*Rickettsia helvetica* organisms were first isolated from *Ixodes ricinus* ticks in Switzerland and were considered to be a new nonpathogenic species of the spotted fever group (SFG) rickettsiae (*1**,**2*). Recently, *R. helvetica* was linked to acute perimyocarditis, unexplained febrile illness, and sarcoidosis in humans in Europe (*3**–**5*). It is generally accepted that *Dermacentor marginatus* is the main vector of *R. slovaca* and that *I*. *ricinus* is the main vector of *R. helvetica* (*1**,**2**,**6*).

Until now, only in the southern (Mediterranean) part of Croatia have *R*. *conorii*, *R*. *slovaca,* and *R*. *aeschlimannii* been detected in *Rhipicephalus sanguineus*, *D. marginatus,* and *Hyalomma marginatum* ticks, respectively (*7*). Human disease caused by *R. conorii* (Mediterranean spotted fever) has also been described in this region (*8*). No published reports of *R. helvetica* in Croatia are available. In a previous study, antibodies to SFG rickettsiae were found in dogs in the northwestern continental part of the country, where *D. marginatus* and *I. ricinus* ticks are common (*9*). Given the importance of this finding, we set up this study to provide the molecular evidence of the presence of *R. helvetica* and *R. slovaca* in Croatia. Prior to the study, *D. reticulatus* ticks had not been found in Croatia, although they were prevalent in neighboring Hungary (*10*). We used molecular methods to establish whether *D. reticulatus* ticks are also present in Croatia.

## The Study

Using the cloth-dragging method, during March–May 2007 we collected 100 adult *Dermacentor* spp*.* ticks from meadows in 2 different locations near Cakovec, between the Drava and Mura rivers in the central part of Medjimurje County. This area is situated in the northwestern part of Croatia, at 46′′38′N, 16′′43′E, and has a continental climate with an average annual air temperature of 10.4°C at an altitude of 164 m.

To isolate DNA from ticks, we modified the method used by Nilsson et al. (*11*). Before DNA isolation, ticks were disinfected in 70% ethanol and dried. Each tick was mechanically crushed in a Dispomix 25 tube with lysis buffer by using the Dispomix (Medic Tools, Zug, Switzerland). Lysis of each of the crushed tick samples was carried out in a solution of 6.7% sucrose, 0.2% proteinase K, 20 mg/mL lysozyme, and 10 ng/ml RNase A for 16 h at 37°C; 0.5 molar EDTA, and 20% sodium dodecyl sulfate was added and further incubated for 1 h at 37°C. Extraction was performed twice with 80% phenol (1:1, vol:vol) and methylenchloride/ isoamylalcohol (24:1, vol:vol). DNA was precipitated with isopropanol. The DNA-pellet was washed with 70% ethanol and centrifuged at 16.000 × *g* for 15 min. After the ethanol was removed, the pellet was dried at 50°C and dissolved in 50 μL distilled water.

For the detection of *Dermacentor* spp., we used conventional PCR based on ribosomal internal transcribed spacer 2 (ITS2) sequences (*12*). In addition, we developed quantitative real-time PCR for the detection of *R*. *helvetica* based on the *ompB* gene and *R*. *slovaca* based on the *ompA* gene. Primer and probe sequences are shown in Table 1. *Borrelia* DNA was investigated with real-time PCR described by Schwaiger et al. (*13*).

**Table 1:** Primers and probes designed for real-time PCR*

Species	Primers and probe	Sequence (5′ → 3′)
*Rickettsia helvetica*	*ompB*_forward	GATTTCGACGGTAAAATTACC
	*ompB*_reverse	GCTACCGATATTACCTACAG
	*ompB*_probe†	ACTCTACTGCTACAAGTATGGTTGCTACAG
*R. slovaca*	*ompA*_forward	GTGATAATGTTGGCAATAATAATTGG
	*ompA*_reverse	CTCTCGTTATAATCGAACCAAC
	*ompA*_probe†	CAGCAGGAGTACCATTAGCTACCCCTCC
*Dermacentor* spp.	ITS_forward	GTGCGTCCGTCGACTCGTTTTGA
	ITS_reverse	ACGGCGGACTACGACGGAATGC

*Amplified products: 162 bp (*R. helvetica*); 228 bp (*R. slovaca)*; 646 bp *(D. reticulatus)*; standard curve: slope; Y-intercept and correlation coefficient:  –3.634, 40.129, 0.9937 (*R. helvetica*); –0.23, 42.82, 0.9942 (*R. slovaca*); ITS, internal transcribed spacer. †The TaqMan probes were labeled with the fluorescent dyes FAM at the 5' end and TAMRA as quencher at the 3' end.

All PCR-derived products generated from ticks (11 *ompB*, 3 *ompA,* and 13 ITS2) were sequenced. Sequencing reactions were performed by using a modified Sanger method and the BigDye Terminator Cycle Sequencing Kit version 1.1 (Applied Biosystems, Carlsbad, CA, USA) on an ABI 3730 capillary DNA Analyzer (Applied Biosystems), employing the same primer pairs as for amplification of the PCR products. Sequencing was performed by both Microsynth AG (Balgach, Switzerland) and our laboratory. All sequences were aligned with known sequences by using BLAST (http://blast.ncbi.nim.nih.gov/Blast.cgi).

The sequences of the ITS2 spacer regions obtained from 13 *Dermacentor* spp. ticks infected with *R. helvetica* and *R. slovaca* were 99.8% identical to the corresponding *D*. *reticulatus* ITS2 sequence (S83080) and 85% identical to the corresponding *D*. *marginatus* sequence (S83081). The 646-bp ITS2 spacer fragment is sufficient to discriminate between the *Dermacentor* spp. The consensus sequence of the 13 *D*. *reticulatus* ITS2 spacer regions determined in this study was deposited at the European Molecular Biology Laboratory database under accession no. FM212280.

Results of the identification of *R. helvetica* and *R. slovaca* are shown in Table 2. The amplified *ompB* sequences of *R. helvetica* were 100% identical to the corresponding *ompB* gene of the *R. helvetica* strain C9P9 (AF123725), 92% identical to “Candidatus R. hoogstraalii” (EF629536), 89% identical to *R*. *asiatica* (DQ110870), 84% identical to *R*. *rhipicephali* (AF123719), and 83.3 % identical to *R*. *raoultii* (EU036984, DQ365798, DQ365797). The amplified *ompA* sequences were 100% identical to the corresponding *ompA* gene of *R. slovaca* and showed 2- to 12-bp differences to the corresponding *ompA* sequences of other *Rickettsia* spp.

**Table 2:** Identification of *Dermacentor,* *Rickettsia*, and *Borrelia* species by molecular methods

Species	Identification method	Targeting sequence	Confirmation	Results
*Dermacentor* spp.	PCR	ITS2 (646 bp)	Sequencing	13/13 (100%) positive;* 99.8% identical to *D*. *reticulatus* ITS2 sequence (S83080)
*R*. *helvetica*	Real-time PCR	*ompB* (162 bp)	Sequencing	11/100 (11%) positive;† 100% identical to *R*. *helvetica* strain C9P9 (AF123725)
*R*. *slovaca*	Real-time PCR	*ompA* (228 bp)	Sequencing	3/100 (3%) positive;† 100% identical to *R*. *slovaca* strains‡
*B*. *burgdorferi*	Real-time PCR	*flaB* (p41)	Not done (PCR negative)	0/100 positive; not detected in *Dermacento*r spp. ticks§

*Only infected ticks (13 ticks) were identified by molecular methods (PCR and sequencing). †100 ticks were analyzed; 10 were positive for *R*. *helvetica* only, 2 for *R*. *slovaca*; 1 was co-infected with *R. helvetica* and *R. slovaca*. ‡ GenBank accession nos. EU622810, DQ649052, DQ649051, DQ649050, DQ649049, DQ649048, DQ649047, DQ649046, DQ649045, DQ649030, DQ649029, Q649027, DQ649054, DQ649053, and U43808. §DNA of *B*. *burgdorferi* DSM 4680 and *B*. *afzelii* DSM 10508 were used as positive controls.

In summary, *R. helvetica* DNA was detected in 10 of 100 *D. reticulatus* ticks, and the pathogen loads ranged from 380 to 1,700 copies per tick. *R. slovaca* DNA was found in 2 of 100 *D. reticulatus* ticks with copy numbers of 400 and 460. One *D. reticulatus* tick was co-infected with *R. helvetica* (410 copies) and *R. slovaca* (20,000 copies). No *Borrelia burgdorferi* DNA was found in *D.* *reticulatus* ticks.

## Conclusions

Scientific literature supports the premise that *D. marginatus* ticks are the main vector of *R. slovaca* and that *I*. *ricinus* ticks are the main vector of *R. helvetica* (*1**,**2**,**6*). *R. slovaca* was also detected in *D*. *reticulatus* ticks (*6**,**14*). Additionally, the DNA of *R*. *raoultii* strain Marne, which is well separated from *R. helvetica* according to phylogenetic analyses of 16S rDNA sequences, was also detected in *D*. *reticulatus* ticks (*14**,**15*).

Previous studies showed that *D. marginatus* ticks are common in Croatia, and *R. slovaca* was identified in 36.8% of *D. marginatus* ticks collected in the southern part of the country (*7*). Further, *R. helvetica* as well as *D. reticulatus* ticks have never been detected in Croatia. In our study, 2% of *D. reticulatus* ticks were infected by *R. slovaca*, 10% were positive for *R. helvetica*, and 1% (1 tick) was co-infected by both pathogens. Our findings may explain the high seroprevalence (20.7%) of SFG antibodies in dogs detected in a previous study in the continental part of Croatia that is *R. conorii* free (*9*). This study suggests that these antibodies to SFG rickettsiae are presumably related to *R. helvetica* and *R. slovaca* infections, which can be transmitted by the same tick vector.

Because *D. reticulatus* is the second most common tick species occurring in all 16 counties of neighboring Hungary, we believe our findings point to an enlargement of its distribution area (*10*). Visual identification of *Dermacentor* spp. ticks has traditionally been confirmed on the basis of morphologic features. Because *D. marginatus* and *D. reticulatus* exhibit overlapping phenotypes, this means of identification can be very difficult (*12*). Therefore, we cannot exclude the possibility that *D. reticulatus* ticks were frequently misinterpreted as *D. marginatus.* Our study shows that the identification problem can be solved through use of molecular biology techniques.

We provide molecular evidence of the existence of *D. reticulatus* ticks in Croatia. Our results expand the knowledge of *R. helvetica* hosts*.* *D. reticulatus* ticks occur at far more sites than previously known and thus have probably expanded their habitats. Our data point out the need for further studies on the epidemiology of *R. helvetica* and other SFG rickettsiae in Croatia as well as their association with infections in humans and animals.
